# Global serum glycoform profiling for the investigation of dystroglycanopathies & Congenital Disorders of Glycosylation

**DOI:** 10.1016/j.ymgmr.2016.03.002

**Published:** 2016-04-17

**Authors:** Wendy E. Heywood, Emily Bliss, Philippa Mills, Jale Yuzugulen, Gabriela Carreno, Peter T. Clayton, Francesco Muntoni, Viki C. Worthington, Silvia Torelli, Neil J. Sebire, Kevin Mills, Stephanie Grunewald

**Affiliations:** a Centre for Inborn Errors of Metabolism, Great Ormond Street Hospital, Great Ormond Street, London WC1N 3JH, UK; b Centre for Translational Omics, UCL Institute of Child Health & Great Ormond Street Hospital NHS Foundation Trust, London WC1N 1EH, UK; c Dubowitz Neuromuscular Centre, UCL Institute of Child Health and Great Ormond Street Hospital, NHS Foundation Trust, London WC1N 1EH, UK; d Neuroimmunology & CSF Laboratory, Institute of Neurology, Queen Square, London WC1N 3BG, UK; e Histopathology Department Great Ormond Street Hospital, NHS Foundation Trust, London WC1N 1EH, UK

**Keywords:** 2D DIGE, 2-dimensional differential gel expression, CDG, Congenital Disorders of Glycosylation, TFN, transferrin, MD, muscular dystrophy, IEF, isoelectric focusing, MW, molecular weight, COG, conserved oligomeric golgi, Congenital Disorders of Glycosylation, Dystroglycanopathies, 2D DIGE, C1-esterase inhibitor, Glycoproteome, α1-Antitrypsin

## Abstract

The Congenital Disorders of Glycosylation (CDG) are an expanding group of genetic disorders which encompass a spectrum of glycosylation defects of protein and lipids, including *N*- & *O*-linked defects and among the latter are the muscular dystroglycanopathies (MD). Initial screening of CDG is usually based on the investigation of the glycoproteins transferrin, and/or apolipoprotein CIII. These biomarkers do not always detect complex or subtle defects present in older patients, therefore there is a need to investigate additional glycoproteins in some cases. We describe a sensitive 2D-Differential Gel Electrophoresis (DIGE) method that provides a global analysis of the serum glycoproteome. Patient samples from PMM2-CDG (*n* = 5), CDG-II (*n* = 7), MD and known complex *N*- & *O*-linked glycosylation defects (*n* = 3) were analysed by 2D DIGE. Using this technique we demonstrated characteristic changes in mass and charge in PMM2-CDG and in charge in CDG-II for α1-antitrypsin, α1-antichymotrypsin, α2-HS-glycoprotein, ceruloplasmin, and α1-acid glycoproteins 1&2. Analysis of the samples with known *N*- & *O*-linked defects identified a lower molecular weight glycoform of C1-esterase inhibitor that was not observed in the *N*-linked glycosylation disorders indicating the change is likely due to affected *O*-glycosylation. In addition, we could identify abnormal serum glycoproteins in LARGE and B3GALNT2-deficient muscular dystrophies. The results demonstrate that the glycoform pattern is varied for some CDG patients not all glycoproteins are consistently affected and analysis of more than one protein in complex cases is warranted. 2D DIGE is an ideal method to investigate the global glycoproteome and is a potentially powerful tool and secondary test for aiding the complex diagnosis and sub classification of CDG. The technique has further potential in monitoring patients for future treatment strategies. In an era of shifting emphasis from gel- to mass-spectral based proteomics techniques, we demonstrate that 2D-DIGE remains a powerful method for studying global changes in post-translational modifications of proteins.

## 1 Introduction

Congenital Disorders of Glycosylation (CDG) are a group of rare inborn errors of metabolism and include some forms of dystroglycanopathies. They are an expanding family of predominantly autosomal recessive multi-systemic disorders [1]. Since glycosylation is a complex biological process involving many pathways, any defect may result in defective glycosylation. Hence there are > 60 distinct disorders identified to date [2], [3]. These are sub-grouped into defects of protein *N*-glycosylation, protein *O*-glycosylation, lipid glycosylation, GDP-anchor glycosylation and multiple glycosylation defects [4]. CDG have a very broad range of clinical symptoms that can involve many organs with a wide spectrum of clinical severity [5] making initial clinical assessment challenging. The most common form of CDG results primarily from defects in the phosphomannomutase 2 gene (termed PMM2-CDG) this disrupts early steps in the glycan assembly and attachment of glycans to proteins resulting in the complete absence of *N*-linked glycans on some glycosylation sites (macroheterogeneity). CDG type II disorders are a result of defective remodeling of glycans resulting in truncated and abnormal glycan structures (microheterogeneity). Several CDG-II causing mutations are in genes involved in the Conserved Oligomeric Golgi (COG) complex [6].

*O*-glycosylation is an even more complex process [7]. The clinical spectrum within a single disorder and among the different inborn errors of *O*-glycan metabolism is vast and the disorders described to date may represent only a subset [8]. One such class of *O*-linked CDG are the dystroglycanopathies [9]. Alpha dystroglycanopathy is a large glycoprotein of approximately 156 kDa in skeletal muscle and is heavily N and *O*-glycosylated as well as *O*-mannosylated [10]. Many of the described mutations for muscular dystrophy affect the *O*-glycosylation pathways for the biosynthesis of α-dystroglycan and new types of CDG have been described that result in defects in the *O*-mannosyl glycosylation pathway [11], [12], [13], [14].

Traditionally the isoelectric focusing (IEF) pattern of serum transferrin is used to detect disorders of protein *N*-glycosylation and is usually the first step in screening for CDG. Transferrin is a 79 kDa serum glycoprotein with two *N*-linked glycans and is highly abundant in serum. A positive transferrin result needs to be followed up by further enzyme and/or genetic testing to identify the specific type of CDG. A similar IEF test to help diagnose *O*-linked disorders is performed on apolipoprotein-CIII which has a single *O*-linked glycan which only captures abnormalities of core-1mucin type *O*-glycosylation [15]. Both of these tests require specialist laboratories to run and interpret the results for a conclusive diagnosis.

While serum transferrin IEF has proven to be a useful test for defective *N*-glycosylation [5], it only identifies differences in charge and lacks information on changes in molecular weight (MW). In addition, secondary glycosylation disorders including galactosaemia, hereditary fructose intolerance, bacterial infections [16] and transferrin polymorphisms must be excluded before considering a diagnosis of CDG. Other methods of CDG investigation include isoelectric focusing of other glycoproteins or investigation of the total glycan profile from glycans removed from the glycoproteins using PNGase F digestion and MALDI analysis [17]. There are a number of suspected CDG cases which do not have altered transferrin IEF profiles or for whom traditional tests provide inconclusive results. Therefore a secondary test to confirm the frontline IEF tests currently used for complex cases is needed. Therefore we have optimised the 2-Dimensional –Difference Gel Electrophoresis (2D-DIGE) method [18] to examine the global serum glycoproteome. 2D-DIGE is an ideal method to investigate changes in post-translational modifications of proteins since multiple samples can be run simultaneously, eliminating gel-to-gel variations and allowing for direct overlaid comparison. This is particularly useful in interpreting subtle or complex CDG type II cases in which the changes in IEF may be subtle. The ability to include confirmed PMM2-CDG and CDG-II samples as internal standards makes interpretation more reliable. Subtle changes in charge and molecular weight can also be observed allowing the detection of a range of defects in post-translational modifications including those of glycosylation that traditional 2D PAGE methods would not detect. To our knowledge this is the first time this technique has been applied to such a large cohort of diverse CDG patient samples. We observed glycoform profile changes for *N*-linked and *O*-linked disorders in looking at various *N*-glycosylated glycoproteins such as α1-antitrypsin, α-1-antichymotrypsin, and α1-acid glycoproteins 1 & 2 and *N*- and *O*-glycosylated α2-HS-glycoprotein and C1-esterase inhibitor.

## 2 Materials and methods

### 2.1 Patient samples

The study was approved by the local NHS Research Ethics committee (London-Bloomsbury) ref: 12/LO/0905. Samples used in this study were anonymized surplus serum remaining from routine diagnostic analysis and were stored and used for the purpose of improving methods for CDG diagnosis. Serum samples were obtained after separation from the cells by centrifugation (3000 *g*/10 min). All samples were stored frozen at − 80 °C prior to analysis.

Patient age-range at sampling was between four months and five years. CDG patients included were diagnosed and treated at Great Ormond Street Hospital Metabolic Unit. Muscular dystrophy patients were diagnosed by the Dubowitz Neuromuscular Centre team at Great Ormond Street Hospital and previously, at the Hammersmith Hospital, London.

Table 1 summarises the disease samples used in this study and details the defect(s) where known. Briefly, five genetically confirmed PMM2-CDG and seven CDG patients with a type II transferrin IEF pattern were included, as was serum from three muscular dystrophy patients; one with LARGE mutation; one with a B3GALNT2 mutation and one an unexplained dystroglycanopathy. Three patients with *N*- & *O*-linked glycosylation defects and two patients with inconclusive transferrin or apolipoprotein CIII IEF profiles and with suspected CDG disorders were analysed to test the method developed. Ten age matched anonymized control samples were selected from surplus serum of patients visiting GOSH for other purposes.

### 2.2 2-Dimensional difference gel electrophoresis (2D DIGE)

For each sample 40 μl of serum was depleted for albumin and IgG using a ProteoPrep Albumin/IgG Depletion Kit (Sigma-Aldrich, Dorset, U.K.). Depleted protein was eluted in 50 mM ammonium bicarbonate buffer pH 7.8 and assayed for protein concentration using the bicinchoninic acid assay kit (Sigma-Aldrich, Dorset, U.K.). Protein was aliquoted into required amounts and freeze dried.

Freeze-dried serum protein (50 μg) was labeled with Cy-Dye DIGE Fluor minimal dye (GE Healthcare) at a concentration of 200 pmol of dye/50 μg of protein. Samples were labeled with Cy3, Cy5, or Cy2. Control protein consisted of pooled sera from five normal subjects, this was used as the internal standard run on all gels and represent the ‘normal’ glycoform profile to compare patient samples with. All three labeled samples, (pooled control and two patients), were combined and resolved on one gel. Combined samples were added to IPG strip rehydration buffer (7 M Urea, 2 M Thiourea, 2% CHAPS, 50 mM DTE and 1% 4–7 IPG buffer) with pH 4–7 or 3–6.5 immobiline drystrips. IEF strips were left at room temperature for 12 h to rehydrate prior to analysis and run the following day on an IPG multiphor (GE Healthcare). Each IEF strip was focused for between 90 and 100,000 V h. After IEF all strips were flash frozen using liquid nitrogen and stored at − 80 °C. IEF strips were re-equilibrated prior to separation by PAGE using a method previously described [19]. PAGE was carried out on 10% and 15% acrylamide gels using piperazine diacrylamide as the cross-linker to improve the resolution of proteins. Gels were scanned in a Typhoon Variable Mode Imager (GE Healthcare) using appropriate lasers and filters with photomultiplier (PMT) voltage between 550 and 600 V. 2D DIGE gel images were analysed using Progenesis Same Spots software version 4.2 (Non-Linear Dynamics, Ltd). All protein identities were confirmed by in-gel digestion of proteins.

### 2.3 In-gel proteolytic digestion and identification of proteins

In-gel digestion of proteins was performed according to published methods [19], [20]. All analyses were performed as described previously [21] using a nano-Acquity UPLC and QTOF Premier mass spectrometer (Waters Corporation, Manchester, UK).

Data were analysed using ProteinLynx Global Server (PLGS) version 2.4 (Waters Corporation, Manchester, UK) with a downloaded Uniprot Human Proteome database. Search settings allowed a minimum three ion matches per peptide, seven ion matches and three peptides matches per protein. Modifications taken into account included up to three missed cleavage sites for trypsin with carboxymethylated cysteine as a fixed modification and oxidation of methionine as a variable modification. Only proteins with a PLGS score > 95% confidence were considered. Mass spectrometry data is provided in the Supplementary data Table S1.

### 2.4 Western Blot analysis

Dilutions of serum samples were made in Phosphate Buffered Saline (PBS), pH 7.2 and the equivalent of 0.75 μl of serum was separated by 10% SDS-PAGE under reducing conditions. Proteins were blotted onto Hybond-LFP PVDF membrane (GE Healthcare, Amersham, UK). Blots were blocked for 1 h in 5% *w*/*v* non-fat dried milk in PBS. Membranes were incubated for 2 h at room temperature with 1 μg/ml mouse monoclonal antibody to C1 esterase inhibitor (Abcam plc, Cambridge, UK), at a 1:2000 dilution. Blots were washed six times for 10 min with 0.1% Tween 20 in PBS before being incubated for 1 h with anti-mouse HRP labeled secondary antibody 1:2500 dilution. Blots were washed and dried and subjected to ECL (GE Healthcare, Amersham UK).

## 3 Results and discussion

### 3.1 Altered glycoproteins observed in *N*-linked glycosylation disorders

**Fig. 1:**
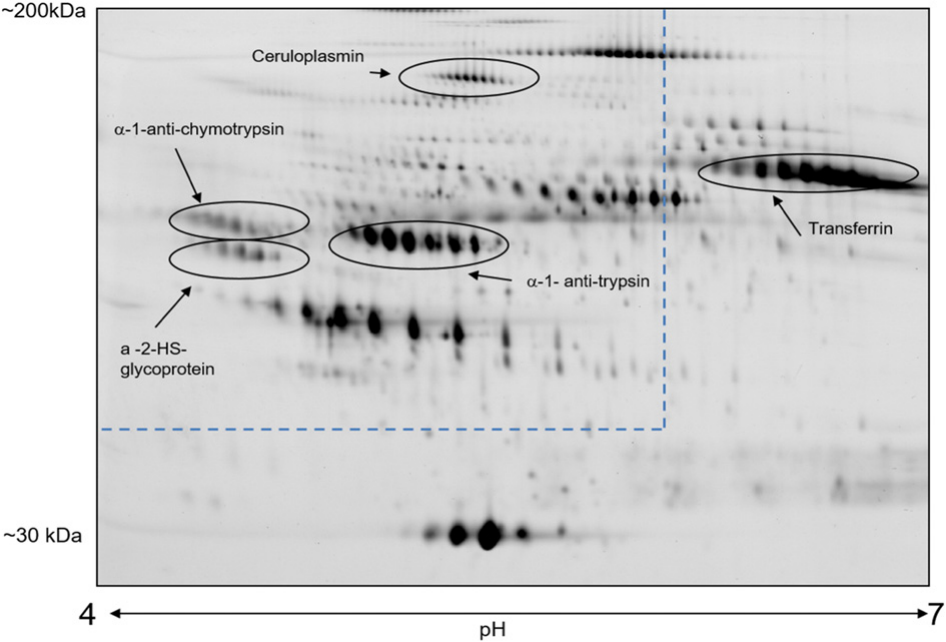
Representative 15% 2D PAGE of albumin and IgG depleted serum. Highlighted proteins that had detectable changes in charge and mass in PMM2-CDG and CDG-II samples. The area defined by dashed lines equates to the optimised higher resolution area for glycoform analysis which was in the 3–5.6 pH range with a 10% acrylamide PAGE for proteins 40–200 kDa.

The initial 2D DIGE experiment over the pH range 4–7 revealed clear changes in charge and mass for transferrin, α1-antitrypsin, α1-antichymotrypsin, α2-HS-glycoprotein and ceruloplasmin when patient samples were compared to that of the pooled control (Fig. 1).

Analysis of the 2D-DIGE gel over the pH range 4–7 demonstrated that the proteins observed displaying the most changes in glycosylation were observed in those glycoproteins observed in the 3–5 pH range. Therefore, a high resolution and targeted 2D-DIGE method over pH range 3–5.6 was used in all subsequent analyses. Using this technique proteins identified as being more significant diagnostically and more subtle markers of inborn errors of glycosylation included α1-antitrypsin, α1-antichymotrypsin, α2-HS-glycoprotein, C1-esterase inhibitor and ceruloplasmin. Changes in transferrin were observed in the initial analysis in the 4–7 pH range, however after optimising the conditions to look at the 3–5 pH range we were no longer able to observe transferrin as the pI for transferrin is 6.5–7. As transferrin profiles had already been performed and were used for the selection of the samples the presence of transferrin in the global profile analysis was deemed not essential.

### 3.2 Characteristic changes observed in PMM2-CDG and CDG-II

#### 3.2.1 α_1_-Antitrypsin is a glycoprotein with 3 *N*-linked glycans

**Fig. 2:**
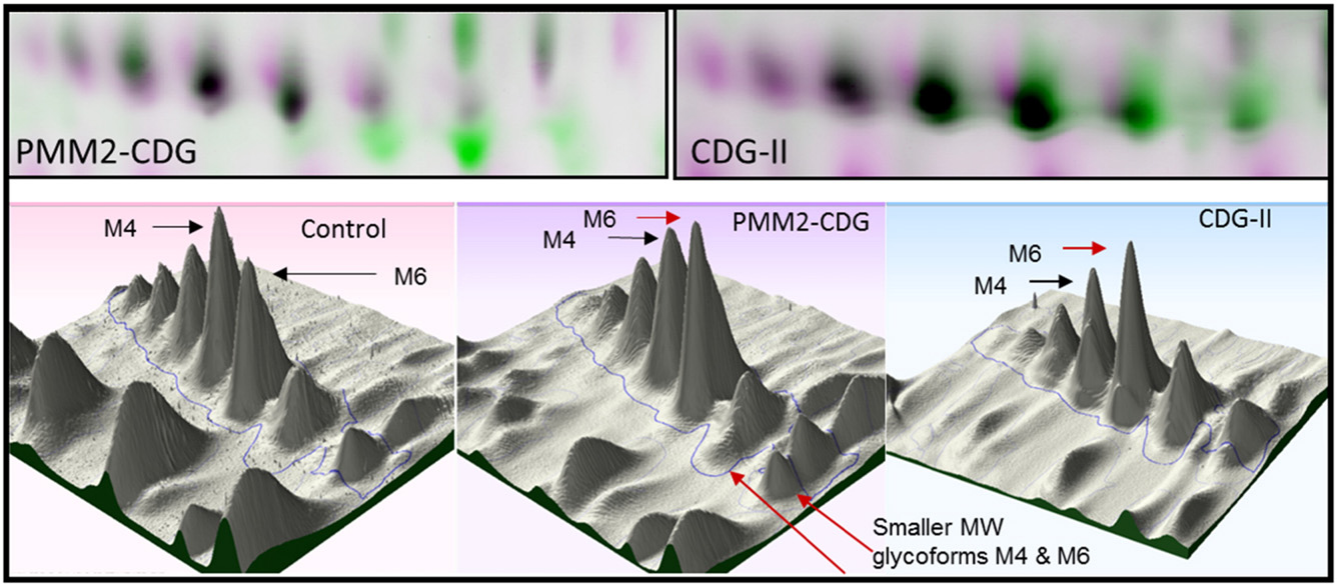
2D DIGE analysis of alpha-1 antitrypsin. Upper panels show overlaid images of α1-antitrypsin from control (pink) and PMM2-CDG and CDG-II patients (green). Underglycosylated M4 and M6 glycoforms of α1-antitrypsin are readily detectable in the PMM2-CDG and are shown in green. Shifts in charge and mass of the glycoforms of the protein can be easily observed. The middle panel shows a 3D schematic of the above images and highlights quantitative changes in the α1-antitrypsin M4 and M6 glycoform peaks.

Analysis over the pH range 3–5.6 demonstrated significant and consistent shifts in pI of the α1-antitrypsin glycoforms for all PMM2-CDG and CDG-II patients (Fig. 2).

Isoelectric focusing of serum α1-antitrypsin leads to the detection of eight bands or glycoforms, known as α1-antitrypsin glycoforms M1 to M8 (anodal-low pH to cathodal-high pH). The bands M4 and M6 are the most abundant glycoforms, making up 40% and 34% of the total serum α1-antitrypsin, respectively, whereas M3 and M5 are present in only trace amounts. All CDG patients tested exhibited a charge shift of the M4 and M6 glycoforms of α1-antitrypsin as described previously [19]. These reduced molecular weight glycoforms were only observed in the PMM2-CDG patients. Analysis of individual α1-antitrypsin glycoforms shown in Fig. 2 were performed using the Progenesis ‘Same Spots’ software (Non Linear Dynamics). Comparison of the spot intensities revealed the most abundant glycoform of α1-antitrypsin to be M4 in a normal profile but in all CDG cases the most abundant was the lesser charged M6 glycoform. This data indicates in all the CDG cases, the presence of truncated glycans resulting in a shift of each glycoform to a higher pI value due to loss of the negatively charged sialic residues. Alpha-1-antitrypsin was also the consistently better glycoprotein biomarker for distinguishing PMM2-CDG from CDG-II using the presence of lower molecular weight glycoforms. The two samples of unknown or suspected CDG-II cases (patients A and Z) which gave inconclusive transferrin IEF results both showed a charge reduction for α1-antitrypsin which was not always observed with transferrin. 2D DIGE analysis revealed low MW glycoforms of α1-antitrypsin indicating PMM2-CDG. This data indicates that α1-antitrypsin is a more sensitive protein or biomarker for diagnosing PMM2-CDG cases than transferrin.

#### 3.2.2 Ceruloplasmin

**Fig. 3:**
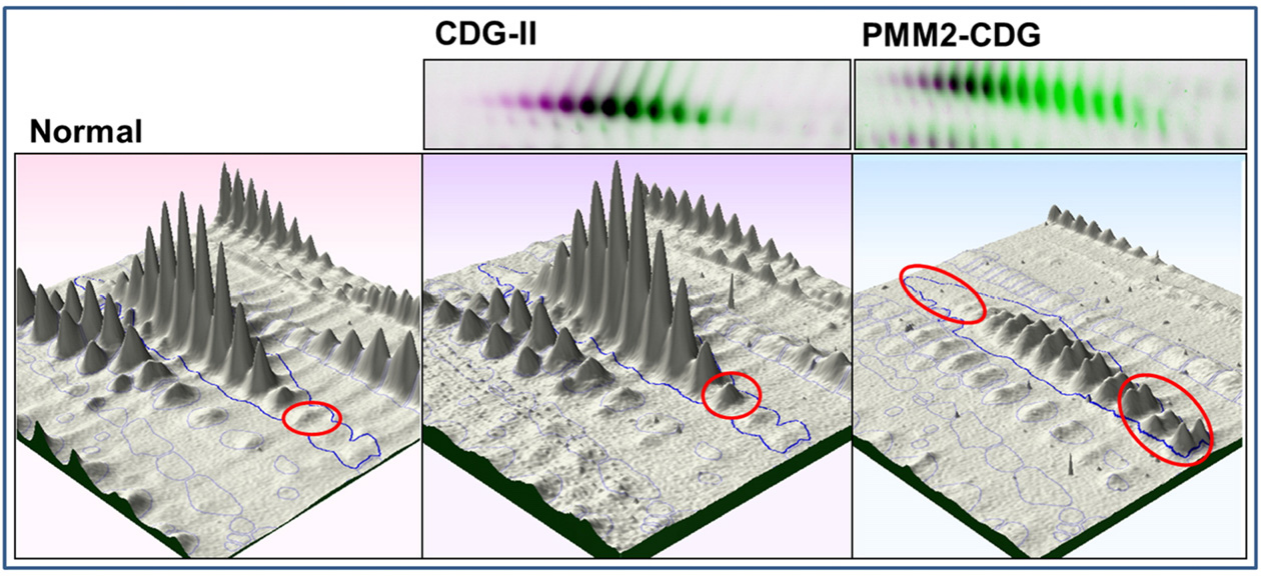
2D DIGE analysis of ceruloplasmin in control, PMM2-CDG and CDG-II. Representative overlaid (top panels) and 3D images of ceruloplasmin illustrating typical changes observed in CDG-II and PMM2-CDG. The overlaid image shows normal pink and overlaid with either CDG as green. CDG-II patients show a subtle charge change as typically seen with many serum glycoproteins which is highlighted by the red ring. The overall concentration, charge and mass change is significantly affected in PMM2-CDG and indicated by the red rings either side of the ceruloplasmin chain.

Ceruloplasmin is a glycoprotein with 6 *N*-linked glycans and is involved in the transport of copper in serum. 2D DIGE analysis revealed ceruloplasmin to be characteristically altered in PMM2-CDG and CDG-II (Fig. 3).

As with other glycoproteins a charge shift is observed in the glycoforms for these patients. The difference between PMM2-CDG and CDG-II can be further differentiated by the degree of charge shift for ceruloplasmin. PMM2-CDG cases showed a profound shift to the cathode or higher pI range (Fig. 3) whilst it is much more subtle in CDG-II. This shift was more pronounced than that observed in α1-antitrypsin and probably results from the greater number of glycans present on the molecule. In PMM2-CDG there appears to be a gradual mass reduction in the lesser charged glycoforms. However, this was a more subtle change in PMM2-CDG than the distinct lower mass glycoforms which were observed for α1-antitrypsin and transferrin. In addition, a significant reduction in the concentration of ceruloplasmin was observed in the PMM2-CDG patients (Fig. 3). This may be due to the larger size of ceruloplasmin which migrates as 133 kDa on this percentage gel (Fig. 1) and the inability to fully differentiate protein molecular weight changes in a gel at this mass range. Previous 2D-PAGE studies have not described altered ceruloplasmin profiles in CDG and to our knowledge this is the first time it has been reported. Detection has probably been enhanced by the removal of albumin which could have obscured ceruloplasmin in earlier serum 2D PAGE studies.

#### 3.2.3 Other glycoproteins demonstrating changes in PI/molecular weight in CDG

Alpha 1 acid glycoproteins 1 & 2 are observed on a 2D-DIGE as 2 protein spots that are very close to one another (Supplementary Fig. S1 panel B). However, this did not affect the identification of changes observed in PMM2-CDG patients which demonstrated a lower molecular weight glycoform whilst analyses of the CDG-II patient serum demonstrating the presence of an extra isoform of higher pI value. This followed the classical shift in pI and/or molecular weight of a glycoprotein expected and observed due to defects in either micro- or macroheterogeneity, as observed in PMM2-CDG and CDG-II patients, respectively. Alpha 1-antichymotrypsin and α2-HS-glycoprotein appear on a 2D gel as 2 chains of similar glycoforms and have similar molecular weights (Supplementary Fig. S1 panel A). Although changes in glycosylation were indicated in each or either glycoprotein it is difficult to say with certainty what the specific changes are due to this close proximity and makes interpretation difficult hence these glycoproteins not ideal to use for assessment of altered glycosylation by this method hence they have been indicated as variable changes in Table 1.

### 3.3 Analysis of serum from patients with both confirmed *N*- & *O*-linked CDG

Most proteins present in serum are primarily *N*-linked glycoproteins and the only glycoproteins with *O*-linked glycans, that can be observed with confidence using 2D DIGE in the optimal pH range 3–6 were α2-HS-glycoprotein (2 complex *N*-linked glycans and 2 *O*-linked glycans) and C1 esterase inhibitor which has 7 *N*-linked glycans (one complex N glycan) and 8 *O*-linked glycans. Therefore we suspected potential *O*-linked disorders could be indicated by changes in glycoforms of these proteins. To investigate this we analysed samples from patients with genetically defined *N*- & *O*-linked disorders or for whom a positive apo-CIII IEF result had been reported previously (Table 1).

**Fig. 4:**
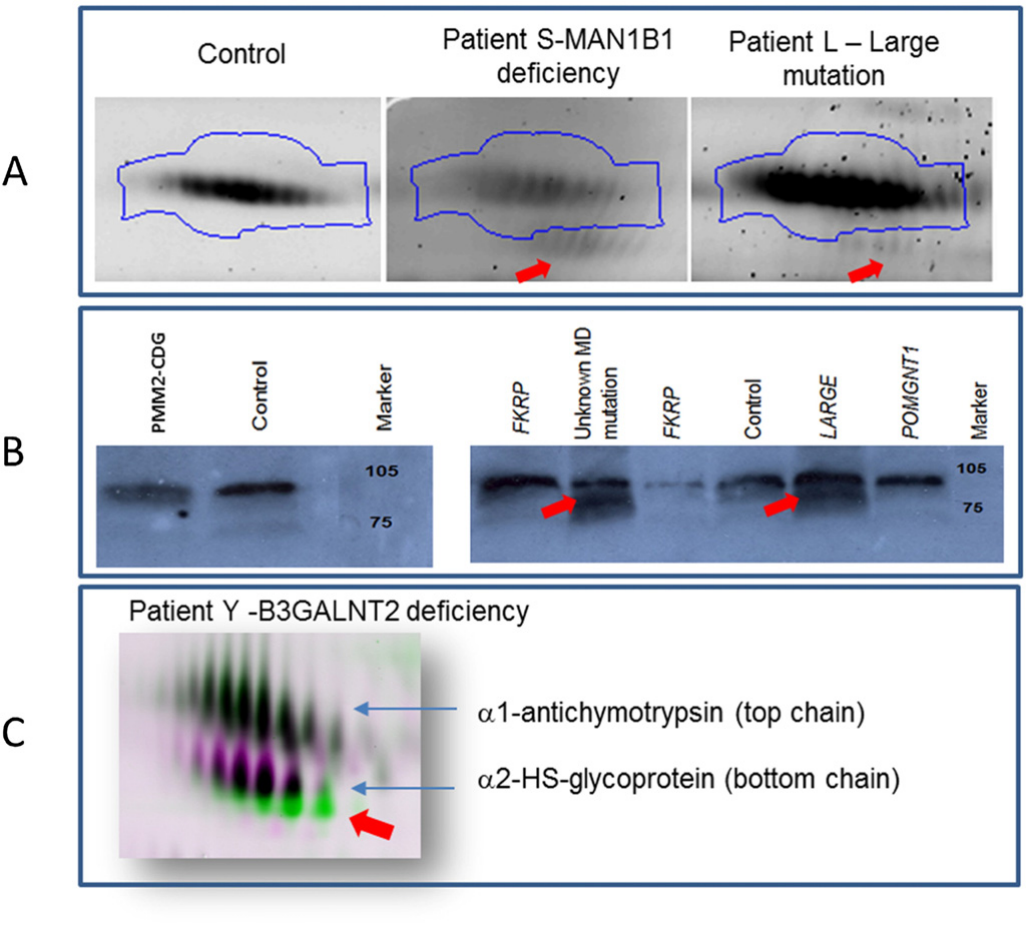
2D DIGE (A & C) and Western Blot (B) analysis of muscular dystrophy patients. Panel A shows 2D DIGE gel images of C1 esterase inhibitor in control, Patient S with an MAN1B1 *N*- & *O*-linked disorder and patient L with a LARGE mutation. A low molecular weight glycoform is observed in both patients. This particular lower molecular weight glycoform is not observed in *N*-linked CDG disorders. Panel B shows 1D western blot images of C1 esterase inhibitor for PMM2-CDG where a lower molecular weight form is observed. This glycoform indicates the degree of mass change for C1 esterase inhibitor in an *N*-linked only disorder. However in the muscular dystrophy patient with a LARGE mutation, an even greater lower molecular weight band is observed (red arrow) as well as in an MD patient with an unknown mutation. No molecular weight glycoforms are observed for other MD mutations FKRP and POMGNT1. Panel C shows an overlaid 2D DIGE image of α1-antichymotrypsin (top chain) and α-2-HS-glycoprotein (bottom chain) of a patient Y an MD patient with a B3GALNT2 mutation. No changes were observed for any *N*-linked proteins in patient Y however a clear lower molecular weight glycoform of α2-HS-glycoprotein is seen. α2-HS-glycoprotein is *N*- & *O*-glycosylated indicating that *O*-glycosylation maybe affected in this patient.

C1 esterase inhibitor shows only a very small molecular weight change for what would be predicted in PMM2-CDG (Supplementary Fig. S1 panel Cb) However as described previously, it is very difficult to confirm at this very low pI range due to breakdown of IEF resolution therefore C1 esterase inhibitor is not an ideal glycoprotein to assess *N*-glycosylation. However patients S, L, J and K did show a much lower molecular weight glycoform (Fig. 4). Fig. 4 shows representative images of C1 esterase inhibitor from a 2D DIGE image of patients with MAN1B1 deficiency and a LARGE mutation. This glycoform was low in abundance and analysis by in-gel digestion and LC-MS/MS confirmed this protein was C1 esterase inhibitor. Further confirmation was obtained using western blotting for C1 esterase inhibitor performed on PMM2-CDG and several confirmed muscular dystrophy patient serum samples (Fig. 4B). The Western blot data confirmed the presence of a smaller MW glycoform (appx 5–10 kDa less) in PMM2-CDG serum as observed by 2D DIGE. An even smaller and fainter MW glycoform of appx 10–20 kDa less was observed for patient L and in patient M with an unknown mutation. Profiles of C1 esterase inhibitor for other MD patient samples with known mutations were however observed to be normal (Fig. 4B). The smaller C1 esterase inhibitor glycoform observed in patient L by Western blotting confirmed the identity of the lower molecular weight glycoform observed by 2D DIGE. This glycoform is not observed in typical PMM2-CDG and CDG-IIx samples and has only been observed in patients with known affected *N*- & *O*-linked glycosylation and apo-CIII positive results (patients S, & K). This indicates strongly that this C1 esterase inhibitor glycoform is present due to altered *O*-glycosylation.

Patient J was diagnosed with a UDP galactose transporter deficiency confirmed by genetic studies [22], which is known to result in reduced transport of galactose to the Golgi apparatus and galactose is required for both *N*- and *O*-glycan assembly. Analysis of transferrin IEF presented with a potential type II pattern. A combined *N*- & *O*-linked disorder was suspected but the apo CIII IEF analyses were not conclusive. Using 2D DIGE we were able to confirm that *N*-linked glycosylation was affected (Table 1), with pI shifts seen typically for CDG type II patterns in α1-antitrypsin, ceruloplasmin and α1-antichymotrypsin. In addition, a lower molecular weight glycoform of C1 esterase inhibitor was also observed indicating an additional defect in *O*-glycosylation. Our analysis shows glycoform profiling of other proteins in particular C1 esterase inhibitor reveals a combined defect in *N*- & *O*-glycosylation is detectable using 2D DIGE analyses of the whole glycoproteome and highlights the need for analysis of more than one glycoprotein as an efficient second tier test in difficult to interpret and complex cases.

Patient K is a cutis laxa patient with a defect in the ATP6V0A2 gene which forms part of the proton channel of V-ATPases [23]. Defects in this proton pump affect the pH of the COG complex which is vital for many functions including *N*- and *O*-glycosylation. Changes in the *N*-linked glycoproteins, ceruloplasmin and α1-antichymotrypsin (Table 1) but not for α1-antitrypsin were observed, thus again highlighting the importance of investigating more than one glycoprotein. Analysis of C1 esterase inhibitor revealed the presence of the smaller molecular weight glycoform indicating affected *O*-glycosylation.

Patient S had a MAN1B1 (alpha-1,2-mannosidase) defect, which has been reported to be localized to the Golgi complex [24]. The precise mechanism of the defect is yet to be elucidated but is thought to affect the glycoprotein synthesis quality control process. Patient S indicated a type II transferrin IEF profile and a positive apo CIII profile. 2D DIGE analysis revealed a CDG type II pattern for α1-antitrypsin, ceruloplasmin but inconclusive observations for α1-antichymotrypsin and α2-HS-glycoprotein. Affected *O*-glycosylation was able to be confirmed by the presence of the smaller C1 esterase inhibitor glycoform. (Table 1, Fig. 4).

This group of three *N*- & *O*-linked disorders overall represent 2 defects in the mechanisms of the assembly of *N*- and *O*-linked glycans (UDP-galactose transporter and MAN1B1 defects) whilst the ATP6V0A2 cutis laxa defect disturbs the optimal environment required for glycosylation in the golgi and hence affects the function of many proteins involved in glycosylation. The serum glycoproteins affected as shown by 2D DIGE do suggest a difference in the way the glycosylation is affected between these classes of disorder as the UDP-galactose transporter and MAN1B1 deficiencies show the same pattern as seen in other CDG-II defects but with additional defective *O*-linked glycosylation. The profile of the cutix laxa patient shows the same apart from a normal profile for α1-antitrypsin indicating a subtler effect. This observation may be useful in the future for initial investigations of complex CDG cases where investigators may look towards defects in genes in either glycan assembly or genes involved in controlling external factors needed for efficient glycosylation (such as golgi pH).

### 3.4 The dystroglycanopathies

Muscular dystrophy patient L had been diagnosed previously with a compound heterozygous mutation in the LARGE gene. The exact function of the LARGE gene remains undetermined but recent reports indicate that it encodes a bifunctional protein with xylosyl- and glucuronyltransferase activities and is required for *O*-mannosylation of α-dystroglycan. LARGE is involved in the synthesis of complex *N*-glycan and mucin type *O*-glycans [25], [26]. 2D DIGE analysis demonstrated a potential PMM2-CDG type profile. Curiously α1-antitrypsin appears to have lower molecular weight glycoforms present but a charge change was not observed. Lower molecular weight glycoforms were observed for ceruloplasmin, α1-antichymotrypsin and α2-HS-glycoprotein suggesting altered *N*-glycosylation but with a very subtle phenotype compared with PMM2-CDG patients. As discussed previously, and shown in Fig. 4, patient L presented with the lower molecular weight C1 esterase inhibitor glycoform confirming affected *O*-glycosylation. Our analysis of the serum glycoproteome of the LARGE mutation patient confirms that many glycoproteins are glycosylated abnormally but not necessarily in a characteristic fashion as typical PMM2-CDG and CDG-II.

Patient Y had B3GALNT2 mutation. Mutations in this gene were recently identified in a group of muscular dystrophy patients with severe structural brain involvement. B3GALNT2 transfers *N*-acetyl galactosamine (GalNAc) in a b-1,3 linkage to *N*-acetyl glucosamine (GlcNAc) and the specific role of this enzyme in α-dystroglycan glycosylation was recently described [27]. As with patient L, 2D DIGE analysis also did not reveal a profile typical to PMM2-CDG or CDG-II with patient Y. However a subtle anodic charge shift and increased MW change was observed for α1-antitrypsin which could potentially be due to increased fucosylation [28]. Other glycoproteins appeared not to be affected apart from α2-HS-glycoprotein which exhibited a distinct reduction in molecular weight (Fig. 4C). Unfortunately the analysis of C1 esterase inhibitor was inconclusive for patient Y. As α2-HS-glycoprotein is the only other *O*-glycosylated protein in the pH range investigated it is possible the reduced MW form observed in this patient may be due to affected *O*-glycosylation.

## 4 Discussion and conclusions

This work does not propose replacement of current techniques for the diagnosis of CDG in routine practice (serum IEF of transferrin and apo CIII). However, in complex cases or those with IEF patterns which are difficult to interpret, 2D DIGE has been demonstrated to be an effective tool for the investigation of not only PMM2-CDG, CDG-II and combined *N*- & *O*-linked disorders but also for the investigation of some muscular dystrophies. Analysing multiple glycoproteins has revealed subtle glycosylation effects that may only be identified on lower abundant glycoproteins. For the muscular dystrophies, baseline tests so far mainly rely on muscle biopsy to stain for α-dystroglycan a method that could potentially be complemented by glycoproteome profiling.

MALDI ToF MS is a method commonly used to determine the structure of glycans in CDG patients [29] but is typically used only for *N*-linked glycans. Mass spectrometry has also been applied to the analysis of glycopeptides which can provide information on both site-specific micro and macroheterogeneity [30]. However, development of an MS based test that could be used to profile multiple *N*-linked glycopeptides and *O*-linked glycopeptides is challenging. This is primarily due to the size of glycopeptides which are too large for most commercial mass spectrometers used in chemical pathology laboratories whose upper mass range is around 2000 Da. The advent of electron-transfer dissociation (ETD), which fragments peptides by a different pathway compared with conventional collision induced dissociation (CID) leaving peptide side-chains (including modifications such as glycans) intact, is promising and analysis of intact *O*-glycopeptides has been reported [31]. However, the ability to detect under glycosylated *O*-glycopeptides in a complex mixture will remain challenging as often the under-glycosylated species are present in low levels in CDG patients therefore analysis of intact glycoproteins by 2D DIGE may be more informative and cost effective.

Analysis of suspected CDG samples using 2D DIGE helped confirm two patients with typical PMM2-CDG profiles (patients A and Z, Table 1). We were also able to show seven patients had normal serum glycoform profiles and were eliminated from having a defect in *N*- or *O*-linked glycosylation (data not shown).

The analysis of 5 patients with molecular genetically confirmed diagnosis of N & O linked CDG and dystrogycanopathies reveals the potential of the method for looking at complex cases of CDG related to different glycosylation pathways. To identify and confirm recognizable patterns further rare complex cases are required to be analysed. 2D DIGE has primarily been used for global differential protein expression and to our knowledge this is the first application of this method to the investigation of glycosylation disorders. Previous studies have utilized silver stained 2D gels to investigate large charge shifts and MW changes. By using 2D DIGE, where control and patient samples can be analysed simultaneously on the same gel, we have been able to observe more subtle changes in other glycoproteins with higher resolution, which has not been reported previously. The technique was improved further by the inclusion of an initial depletion step removing the abundant serum proteins albumin and immunoglobulin G and the selection of a high resolution IEF range for candidate marker glycoporoteins. The incorporation of a depletion step was able to reveal ceruloplasmin as a good marker for *N*-linked glycosylation which has also not been described previously.

In summary, serum glycoform profiling by 2D DIGE has proven to be a second tier useful technique in assessing defective glycosylation. α1-antitrypsin appears to be the ideal protein to distinguish PMM2-CDG and CDG-II patients however as demonstrated on more than one occasion in this study, reduced glycosylation of individual proteins between patients is variable and ideally more than one glycoprotein should be assessed at screening. This method can also not just be applied to serum but also other CDG tissues to identify affected glycoproteins. [32]. Characterising a glycoproteome profile of patients prior to and on treatment will help to get a better understanding in the changes of a plethora of glycoproteins and related clinical observations. 2D DIGE could become a very useful and an informative tool for monitoring the effect of current and future treatments for CDG.

The following are the supplementary data related to this article.

Supplementary Fig. S1 Other affected glycoproteins in CDG. Panel A shows overlaid representative 2D DIGE image of α-1-anti-chymotrypsin (top chain) and α 2-HS-glycoprotein (bottom chain) from a PMM2-CDG patient. Whilst changes were observed in both proteins for PMM2-CDG and CDG-II they were not consistent and were varied within the disease groups making it difficult to interpret. Panel B shows overlaid images of alpha 1 acid glycoproteins 1&2. Mass and charge changes can be observed in both PMM2-CDG and CDG-II but it is apparent the resolution of IEF breaks down at this high acidic pH. Panel C shows images of C1 esterase inhibitor control (a) and PMM2-CDG (b) indicating a small subtle mass change. (c) Shows an overlaid image of a CDG-II patient. Supplementary Fig. S1

Table S1 Protein identifications including number of peptides and coverage from the LC-MS/MS analysis for each protein are provided as supporting information. Table S1

Supplementary data to this article can be found online at http://dx.doi.org/10.1016/j.ymgmr.2016.03.002.

## Figures and Tables

**Table 1:** Summarised changes observed of serum glycoproteins for the investigation of different forms of CDG.

		α1-antitrypsin		Ceruloplasmin		α-1-antichymotrypsin (sup data Fig 1)		α-2-HS-glycoprotein (sup data Fig 1)		C1 esterase inhibitor		
CDG classification	Patient details	Change in charge	MW change	Large charge change	Small charge change	Change in charge	MW change	Charge	MW change	Charge	Larger lower MW glycoform	Smaller lower MW glycoform
PMM2-CDG	All patients had mutations in the PMM2 gene	✓	✓	✓		✓	✓ variable	✓	✓ variable	✓	✓	
CDG-IIx	All patients described as CDG-IIx based on transferrin IEF pattern	✓			✓	✓ variable		✓variable		✓ variable		
*N-* & *O-*linked CDG disorders	K—Cutis laxa; mutation in ATP6V0A2 gene				✓	✓		✓			✓	✓
J—UDP-galactose transporter defect	✓			✓	✓		Inconclusive		✓		✓
S—MAN1B1 deficiency type-II tfn pattern, abnormal ApoCIII-1 profile — hyposialylation	✓			✓	Inconclusive		Inconclusive				✓
Dystroglycanopathies	L—MD: mutation in LARGE gene		✓	✓		✓		✓	✓			✓
Y—MD: muscle eye brain disease due to B3GALNT2 mutations	Increased glycosylation		Normal		Normal			✓			Inconclusive
M—MD: mutation unknown	Not profiled by 2D DIGE		Not profiled by 2D DIGE		Not profiled by 2D DIGE		Not profiled by 2D DIGE				✓(by western blot)
Unknown	A—suspected type II CDG	✓	✓	✓						✓		
Z—inconclusive transferrin IEF	✓	✓	✓								

Tfn = transferrin, MD = muscular dystrophy, ‘charge’ refers to an observed charge shift, ‘MW’ refers to an observed glycoform of reduced molecular weight. Typical observations for PMM2-CDG and CDG-II are made in the top two shaded rows and based on consistent changes observed in 5 PMM2-CDG and 7 CDG-II patients. Observations from other CDG samples are listed below for comparison with typical observations for PMM2-CDG and CDG-II. ✓ Indicates that type of change (charge shift or/and presence of molecular weight glycoform was observed, blank cells indicate that change could not be observed by 2D DiGE for that glycoprotein.
