# Supplementary material for: Global serum glycoform profiling for the investigation of dystroglycanopathies & Congenital Disorders of Glycosylation
Source: Mol Genet Metab Rep. 2016 Apr 17;7:55–62. doi: 10.1016/j.ymgmr.2016.03.002 (PMC4834675; doi:10.1016/j.ymgmr.2016.03.002)
Supplement: Supplementary Fig. S1 — Other affected glycoproteins in CDG. Panel A shows overlaid representative 2D DIGE image of α-1-anti-chymotrypsin (top chain) and α 2-HS-glycoprotein (bottom chain) from a PMM2-CDG patient. Whilst changes were observed in both proteins for PMM2-CDG and CDG-II they were not consistent and were varied within the disease groups making it difficult to interpret. Panel B shows overlaid images of alpha 1 acid glycoproteins 1&2. Mass and charge changes can be observed in both PMM2-CDG and CDG-II but it is apparent the resolution of IEF breaks down at this high acidic pH. Panel C shows images of C1 esterase inhibitor control (a) and PMM2-CDG (b) indicating a small subtle mass change. (c) Shows an overlaid image of a CDG-II patient. [file mmc1.pdf]

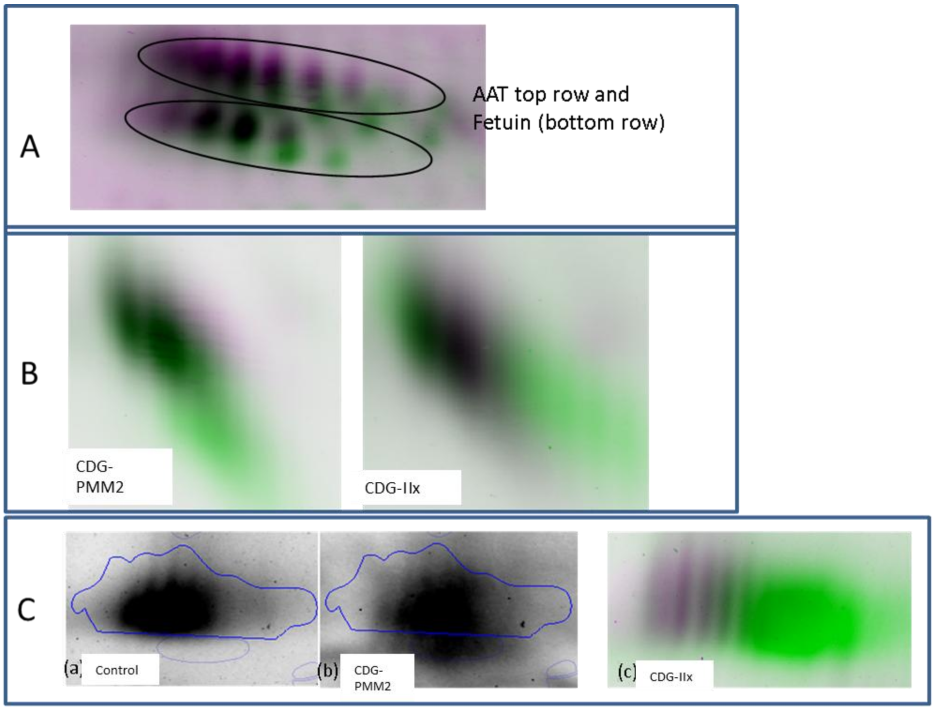

Supplementary figure. Other affected glycoproteins in CDG. Panel A shows overlaid representative 2D DIGE image of α-1-anti-chymotrypsin (top chain) and α 2-HS-glycoprotein (bottom chain) from a PMM2-CDG patient. Whilst changes were observed in both proteins for PMM2-CDG and CDG-II they were not consistent and were varied within the disease groups making it difficult to interpret. Panel B shows overlaid images of alpha 1 acid glycoproteins 1&2. Mass and charge changes can be observed in both PMM2-CDG and CDG-II but it is apparent the resolution of IEF breaks down at this high acidic pH. Panel C shows images of C1 esterase inhibitor control (a) and PMM2-CDG (b) indicating a small subtle mass change. (c) Shows an overlaid image of a CDG-II patient.
